# Incentives and practice improve prospective memory performance in older adults

**DOI:** 10.1007/s40520-025-03127-z

**Published:** 2025-07-09

**Authors:** Marta Menéndez-Granda, Nadine Schmidt, Michael Orth, Sebastian Horn, Matthias Kliegel, Jessica Peter

**Affiliations:** 1 University Hospital of Old Age Psychiatry and Psychotherapy, University of Bern, Bern, Switzerland; 2 Graduate School for Health Sciences, University of Bern, Bern, Switzerland; 3 Department of Psychology, University of Zurich, Zurich, Switzerland; 4 Faculty of Psychology and Educational Sciences, University of Geneva, Geneva, Switzerland; 5 Centre for the Interdisciplinary Study of Gerontology and Vulnerability, University of Geneva, Geneva, Switzerland; 6 Swiss Centre of Expertise in Life Course Research, LIVES Centre, Lausanne and Geneva, Geneva, Switzerland

**Keywords:** Prospective memory, Healthy ageing, Incentive, Financial gains, Financial losses, Practice effect

## Abstract

**Background:**

Prospective memory can decline with age. Incentives or practice may improve it. Previous research indicated that avoiding financial losses may be more appealing for older adults than achieving financial gains.

**Aims:**

In this study, we examined whether financial gains, financial losses, or practice will improve prospective memory performance in older adults. We included *N* = 132 healthy adults (60–80 years old, 35% female).

**Methods:**

We used a between-subject factor ‘group’ (event-based or time-based prospective memory; financial gains or losses) and a within-subject factor ‘practice’ comprising 2 blocks (first or second half of task trials). All participants performed a 1-back task as an ongoing task and a prospective memory task embedded in the 1-back task. We used linear effects modelling to examine whether incentives or practice improved task accuracy, response times, or clock checking.

**Results:**

The prospect of financial losses accelerated event-based prospective memory responses and led to more strategic clock checking in the time-based prospective memory task compared to the prospect of financial gains. In addition, it accelerated ongoing task responses compared to financial gains. Practice, in contrast, only improved event-based prospective memory with no effect on time-based prospective memory.

**Conclusions:**

Our findings highlight the importance of choosing an incentive adjusted to the performance outcome when designing studies that examine the influence of incentives or practice on prospective memory.

**Supplementary Information:**

The online version contains supplementary material available at 10.1007/s40520-025-03127-z.

## Introduction

Prospective memory is the ability to remember to do something in the future. It can be event-based (e.g., do grocery shopping when seeing a supermarket) or time-based (e.g., call someone at 9 p.m.) [1]. Time-based tasks may be more demanding as no external cues trigger the intention to act [2]. Instead, this requires repeated clock-checking to accomplish the intention on time [3, 4, 5]. Event-based tasks were thought to be less demanding as retrieval is triggered by external events [6]. This initial view has been refined, as event-based tasks can also be demanding, depending on task characteristics [7, 8] (e.g., cue focality). The current view, therefore, is that task characteristics rather than task type determine the cognitive efforts needed to accomplish the task. The less external help there is, the more important become internal control processes. With age, the quality of these internal control processes declines and consequently, prospective memory tasks that rely on them become more challenging [1, 3, 4]. Age is, however, no barrier to learning, and it is well known that practice (i.e., either simple repetition or prolonged training) can have a beneficial effect on cognitive performance even in older people, particularly when they are motivated to learn [9]. This is likely true for prospective memory. According to the motivational-cognitive model of prospective memory, future intentions that align with one’s goals are perceived as more relevant and important. Hence, they are encoded, maintained, and retrieved better [10]. This all indicates that one possible way to slow down decline in prospective memory may be by enhancing the motivation to learn [11, 12]. Goals and interests, however, seem to change across the lifespan. While younger adults tend to acquire new skills and to achieve gains, older adults often prefer maintaining their resources and avoiding losses [13, 14]. In addition, they seem to learn better when successful learning means they avoid negative consequences [15]. Prospective memory studies that tried to enhance the motivation to learn by using the prospect of an incentive, found diverging results depending on age. In younger adults, the prospect of financial gains improved event-based prospective memory [16, 17] but not time-based prospective memory [18]. In older adults, avoiding financial losses improved event-based prospective memory more than the prospect of financial gains [13]. Whether avoiding financial losses also improves time-based prospective memory in older adults, has not been tested so far.

Practicing a task may be another way to enhance prospective memory. In younger adults, practicing the estimation of time for 1 h was associated with better time-based prospective memory [19, 20, 21]. In addition, there were little performance differences between younger and older adults when a prospective memory task (i.e., a virtual week task that included both event-based and time-based prospective memory) was repeated [22, 23]. This indicates that repetition helps older adults to perform better, most likely due to familiarization with the task. The time-based task in that study did not require clock monitoring. Instead, participants had to monitor cues that appeared on a screen as a reminder, which resembles an event-based task. Hence, the effect of practice on time-based task performance is still not fully understood. It may be that practice has less influence on time-based task performance, as these tasks are more demanding for older adults [1]. Hence, they may need more time to internalise the structure of the task and to adapt their performance.

In sum, there is evidence to suggest that in older adults, event-based prospective memory can be enhanced by avoiding financial losses or by practice. It is an important question whether this also holds true for time-based prospective memory. Prospective memory failures in daily life are often followed by financial losses (e.g., forgetting to pay a bill on time or forgetting to return a rented item on time may incur extra costs). A better understanding of whether positive or negative consequences modulate prospective memory would help to discern the mechanisms involved in motivational processes of prospective memory. We therefore examined whether incentives (gains or losses) or practice influenced prospective memory performance in healthy older adults. This extends previous research that investigated either practice effects or the influence of incentives on one prospective memory type. We hypothesized that performance improves particularly when participants are incentivized to avoid financial losses. In addition, we expected that practice improves prospective memory performance particularly in the event-based task as this task is less demanding for older adults [1].

## Methods

### Participants

*N* = 150 participants were recruited and tested using an online panel (i.e., Respondi Cologne, Germany). We excluded 18 participants because their ongoing task accuracy was more than 2 standard deviations below the sample mean. Thus, the final sample consisted of 132 adults (35% female; Table 1). All participants were between 60 and 80 years old, fluent in German, and perceived their health status as good. We used a between-subject factor ‘group’ and randomly assigned participants to one of four experimental conditions: (1) Time-based prospective memory– losses, (2) Event-based prospective memory– losses, (3) Time-based prospective memory– gains (4) Event-based prospective memory– gains. In addition, we used a within-subject factor ‘practice’ defined by two blocks of trials. Block 1 comprised the first half of task trials, block 2 the second half of task trials. We evaluated task performance in terms of accuracy and response times for the event-based task and in terms of accuracy and clock monitoring for the time-based task. Since performing two tasks simultaneously (i.e., ongoing task and prospective memory task) can lead to task interference, we also evaluated ongoing task performance in terms of accuracy and response times. We determined practice effects by comparing task performance in two blocks (block 1: first half of the task; block 2: second half of the task).

No data were obtained with which participants could be identified. Hence, all participants took part in the study anonymously and therefore, no ethical approval was required for this study. We informed participants about the purpose of the study and they provided consent by clicking on a button. In addition, their participation was voluntary and they were free to withdraw from it at any time without giving a reason.

### Prospective memory task

**Fig. 1:**
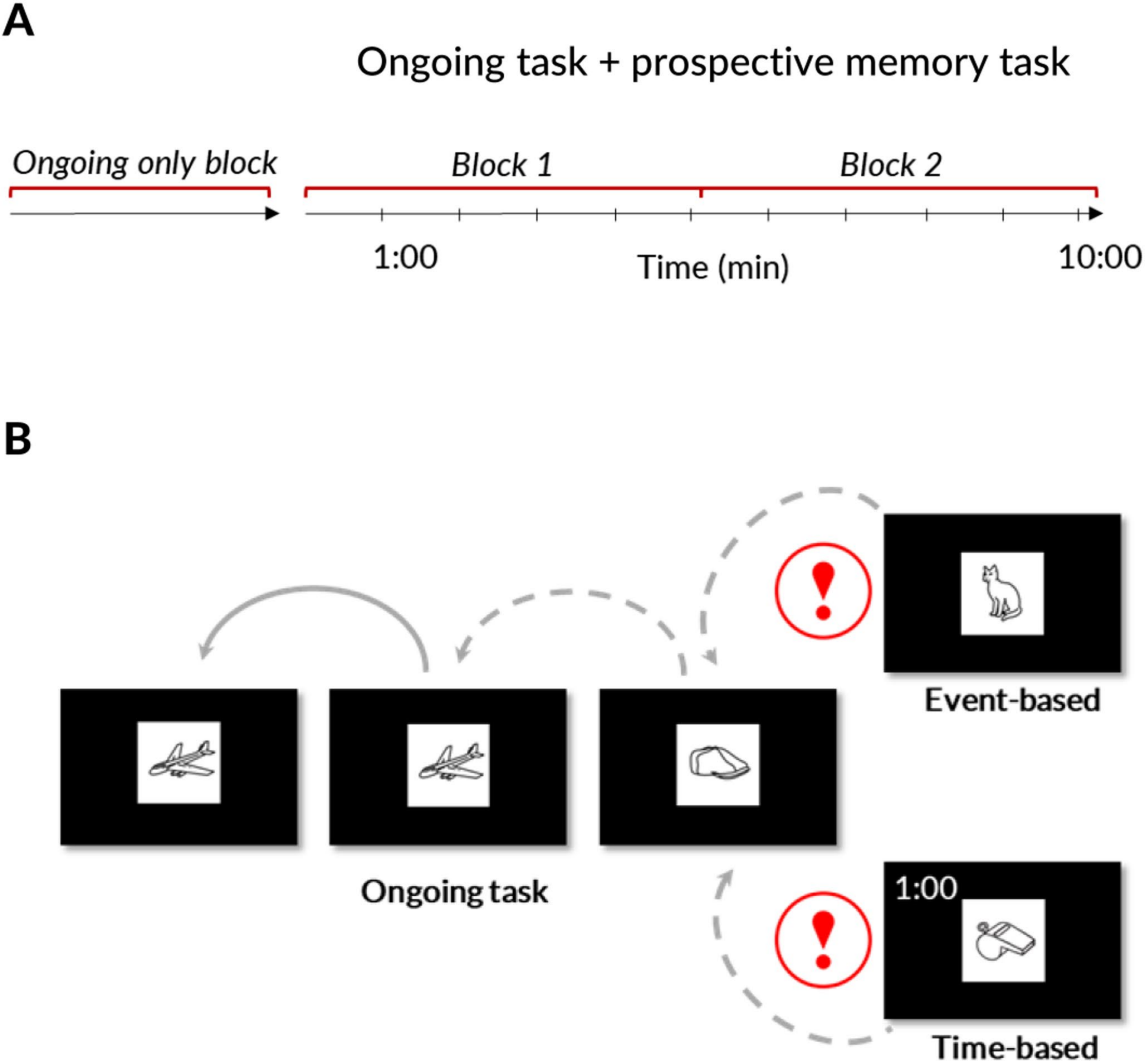
Study procedure (**A**) 1-back task and 1-back task plus prospective memory task. Block 1 and 2 were defined a posteriori to study practice effects (**B**) Illustration of 1-back task and prospective memory task with event-based cue (animal) or time-based cue (one minute). Red exclamation marks indicate prospective memory targets (only for visualization– these are not shown during the experiment). Grey arrows indicate what needs to be done in the 1-back task: a dashed arrow indicates a different image, while a solid arrow indicates identical images

Tasks were presented using PsychoPy v2021.1.2 (Peirce et al., 2019) hosted on Pavlovia, which is a secure online platform for running experiments (https://pavlovia.org/). First, participants had to do a 1-back visual working memory task (i.e., ongoing task, Fig. 1), during which they needed to decide whether the current image was identical with the previous one (left arrow key = yes, right arrow key = no). Each image was presented for 3s with an interstimulus interval of 1s. In the second part, a prospective memory task, adapted from Jäger and Kliegel [5], was added to the ongoing task (Fig. 1). Again, each image was presented for 3s with an interstimulus interval of 1s. For the prospective memory task, participants had to remember to press a button (upper arrow key) whenever an animal appeared (event-based task) or whenever they thought one minute had passed (time-based task). To monitor time, participants could press another button (lower arrow key) for a clock to appear for 3s in the upper left corner of the screen. The 1-back only part included 20 trials. Each prospective memory part included 10 prospective memory targets appearing every minute (i.e., 1:00, 2:00, 3:00,… until 10:00) and 158 1-back trials. Incentives were provided in the second part (i.e., when the prospective memory task was added). In the gain condition, participants were informed that they could earn money depending on their task performance (i.e., 0.50 Swiss Francs per remembered prospective memory target). In the loss condition, participants started with an initial amount of money (i.e., 5 Swiss Francs) which they could lose, depending on the proportion of prospective memory targets that they missed (i.e., 0.50 Swiss Francs per missed target). Since older adults seem to perform particularly well when half of their earnings are donated than when keeping everything for themselves [24], participants in both incentive conditions (i.e., gains and losses) were told that half of their earnings would be donated to an organization of their choice (i.e., Doctors Without Borders, UNICEF, or WWF).

Button presses on the corresponding key (upper arrow) that occurred within ± 2s around target time (for the time-based task) or within 4s after the presentation of an animal (for the event-based task) were considered a prospective memory hit [4, 5]. Response times were defined as the time interval between the presentation of an image (for the 1-back task) or a target (i.e., an animal for the event-based task) and the corresponding button press. For clock monitoring, we calculated how many times the participants pressed a button to make the clock appear within ± 30 s around each target time. Similar to our previous study [25] we divided the 30s before and after each target time into four intervals: T– 30 referred to the interval 30 to 15 s and T– 15 to the interval 15 to 0 s before each target time. The intervals 0 to 15 s and 15 to 30 s after the target time corresponded to T + 15 or T + 30, respectively. We used task accuracy, response times, and clock monitoring for statistical analysis. Response times of accurate answers that were outside ± 2 standard deviations from the sample mean or faster than 150 ms were excluded from statistical analysis according to previous studies [26, 27].

### Statistical analysis

As primary outcome, we examined whether incentives or practice influenced prospective memory performance (accuracy, response times, or clock checking). To test for incentive effects, we used a variable with two levels: gains or losses. To test for practice effects, we divided the prospective memory part of each task into two blocks, each containing five prospective memory targets [28]. Hence, each block had the same length and number of trials. We first examined whether participants answered more accurately depending on task type (event-based or time-based), incentive type (gains or losses), or practice. We fitted a generalized linear mixed model with binomial distribution and logit link function for prospective memory trial accuracy (i.e., for every prospective memory trial in which an animal was displayed or whenever a minute had passed; we used a dichotomous variable with 0 for incorrect/missed or 1 for correct responses). Fixed effects were task type (time-based, event-based), incentive type (gains, losses), and blocks of time (block 1, block 2). Next, we examined whether participants answered faster in the event-based task, depending on incentive type or practice. For this, we fitted a linear mixed model using response time of accurate responses (log-transformed data [29]) as dependent continuous variable and incentive type, and blocks of time as fixed effects. Then, we investigated whether participants checked the clock differently depending on time interval, incentive type, or blocks of time. We fitted a linear mixed model including clock monitoring frequency as dependent continuous variable, and type of incentive, time interval (T– 30, T– 15, T + 15, T + 30), and blocks of time (block 1, block 2) as fixed effects. All three models included participant-specific random effects for the intercept, and we tested for interactions. We used *F*-tests for linear models and X^2^-tests for generalized linear models. We used post-hoc tests of estimated marginal means for significant factors or interactions. For generalized linear models, estimated marginal means were presented as probabilities. For linear mixed models, log response times were back-transformed to estimated means.

As secondary outcomes, we examined whether more frequent clock checking was associated with more accurate time-based responses using Spearman correlations. We used Fisher’s *z*-tests to test whether correlation coefficients differed between gains and losses. Then, we examined whether incentives or practice influenced ongoing task performance (i.e., accuracy or response times) during the prospective memory part of a task, and whether this was different for time-based or event-based prospective memory. We used the same blocks of time as for the prospective memory task. Finally, we examined whether we find any interference when a prospective memory task was added to the ongoing task. We again fitted generalized linear mixed models for ongoing task trial accuracy (i.e., hits and no hits; we used a dichotomous variable with 0 for incorrect/missed responses/false alarms or 1 for correct responses and correct rejections) or linear mixed models for response times. Fixed effects were ongoing task condition (1-back only, 1-back plus prospective memory), task type (event-based, time-based), and incentive type (gains, losses). Again, all models included participant-specific random effects for the intercept, and we tested for interactions.

We used R with RStudio (version 4.1.0) for statistical analyses, with *p* <.05 considered statistically significant. We applied Tukey correction for multiple comparisons. To estimate the appropriate degrees of freedom in our models, we used the Wald and Kenward-Roger approach [30] as implemented in R’s lmerTest package [31] (version 2.0–29). The package lme4 was used to fit and analyse the models [32].

## Results

All groups were similar regarding age, sex, education, income, retirement status, perceived health, and donation frequency (Tables 1, 2 and S1).

### Practice improved event-based task accuracy

**Fig. 2:**
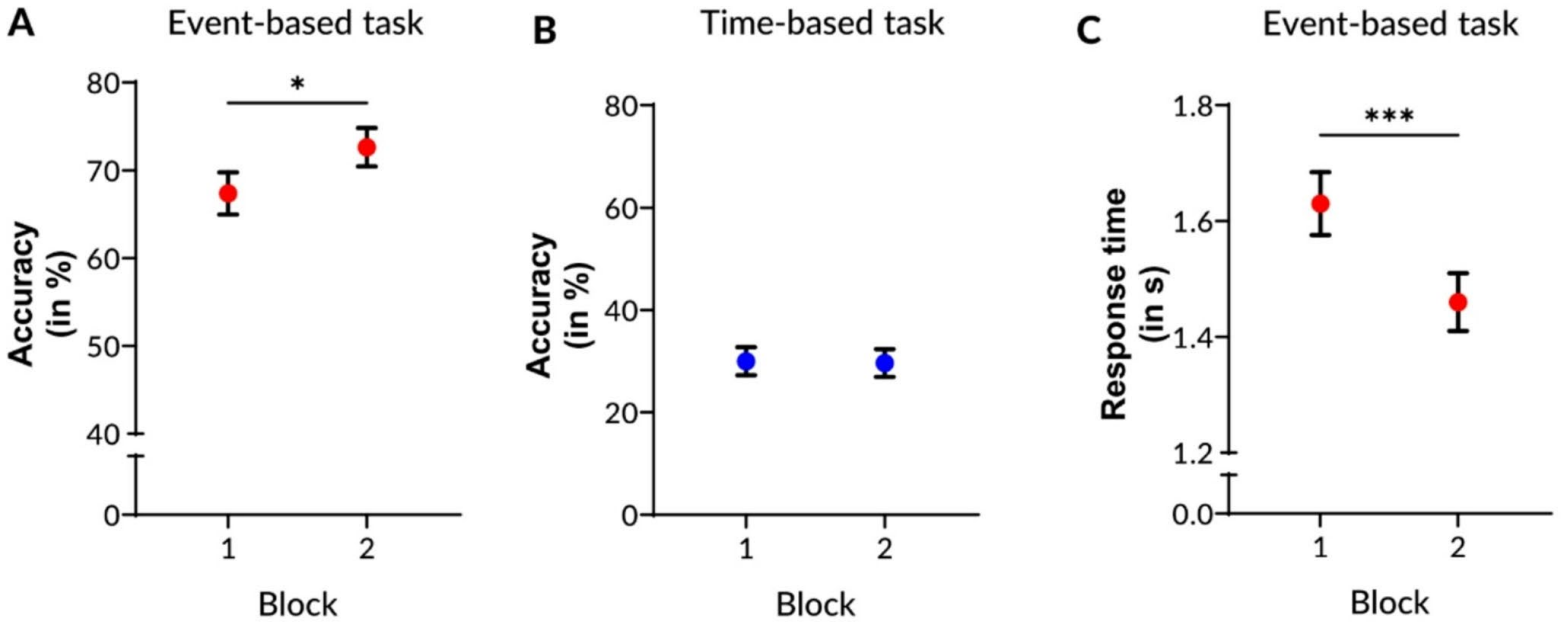
Practice effects in an event-based or time-based prospective memory task. Task performance over time was divided into 2 blocks of 5 min each to test for practice effects in **A**) Task accuracy (probability) in the event-based or **B)** time-based prospective memory task or **B**) Response times (only event-based task). Error bars indicate the standard error of the mean. Significant at *p* <.05^*^ or *p* <.001^***^

We found a significant interaction between task type and blocks of time (*X*^*2*^_(1)_ = 7.16, *p* =.01), as event-based task accuracy improved with practice (β = 1.04, SE = 0.35, *z* = 2.98, *p* =.015; Table 3, Fig. 2a) but time-based task accuracy did not (β = 0.19, SE = 0.32, *z* = 0.6, *p* =.931; Table 3, Fig. 2b). We did not find a significant effect of incentive type (*p* =.94) nor a significant interaction (*p* =.075). These results indicate that for practice to modulate task accuracy, the type of task matters (for comprehensive statistics see Table S2-S6 in the Supplement).

### Participants became faster when avoiding losses or with practice in the event-based task

**Fig. 3:**
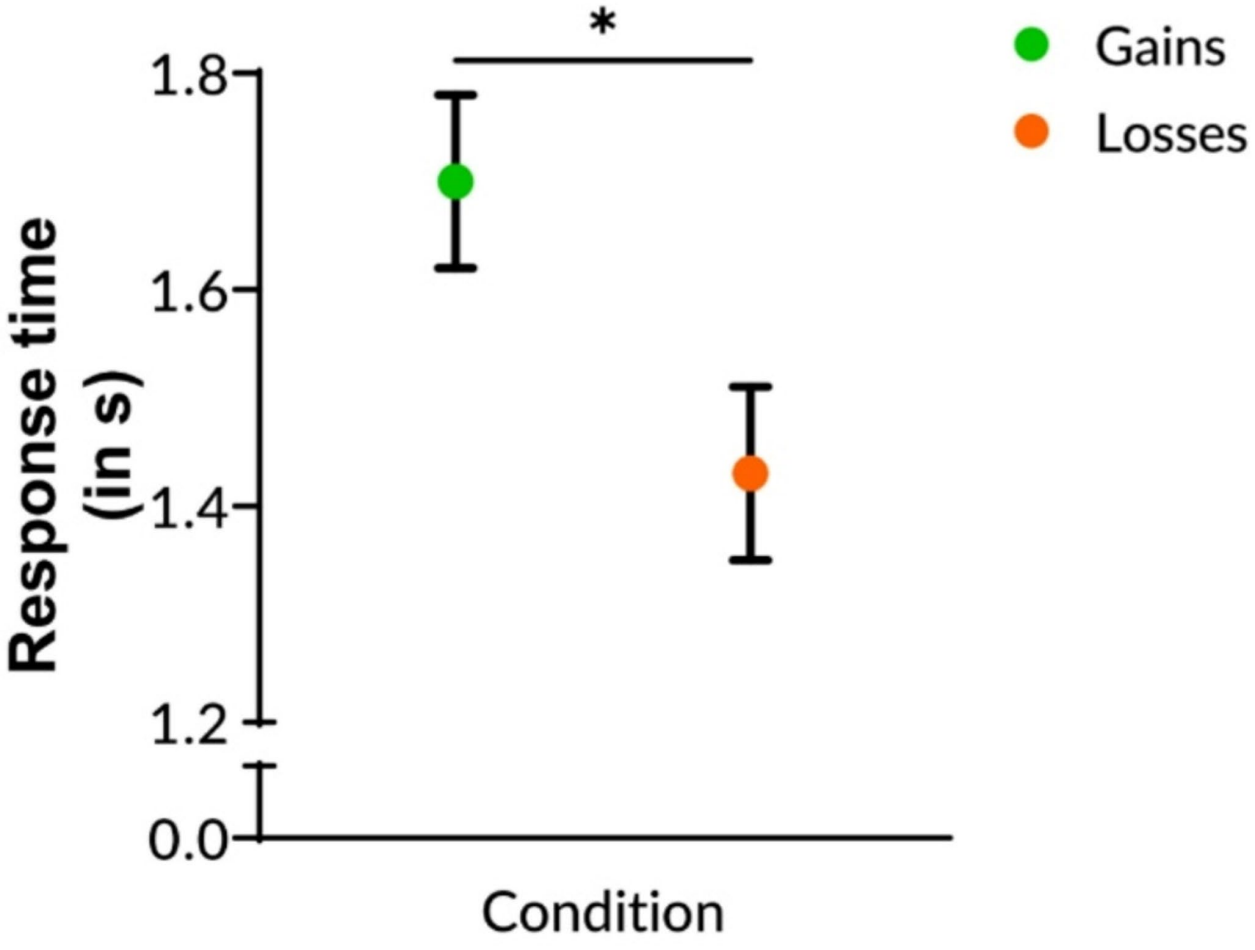
The influence of financial gains or losses on event-based prospective memory response times. Participants were able to achieve financial gains or to avoid financial losses. Error bars indicate the standard error of the mean. Significant at *p* <.05^*^

We found a significant practice effect (*F*_(1, 442.95)_ = 24.18, *p* <.001) as participants responded faster in block 2 than in block 1 (Table 3, Fig. 2c; Tables S7 and S9 in the Supplement). In addition, we found a significant effect of incentive type (*F*_(1, 55.95)_ = 5.57, *p* =.022), as participants responded faster in the loss condition than in the gain condition (Table 3, Fig. 3a; Table S8). This indicates that faster responses in an event-based task can either be achieved by practice or by loss-related incentives.

### Participants checked the clock more strategically when they avoided financial losses

**Fig. 4:**
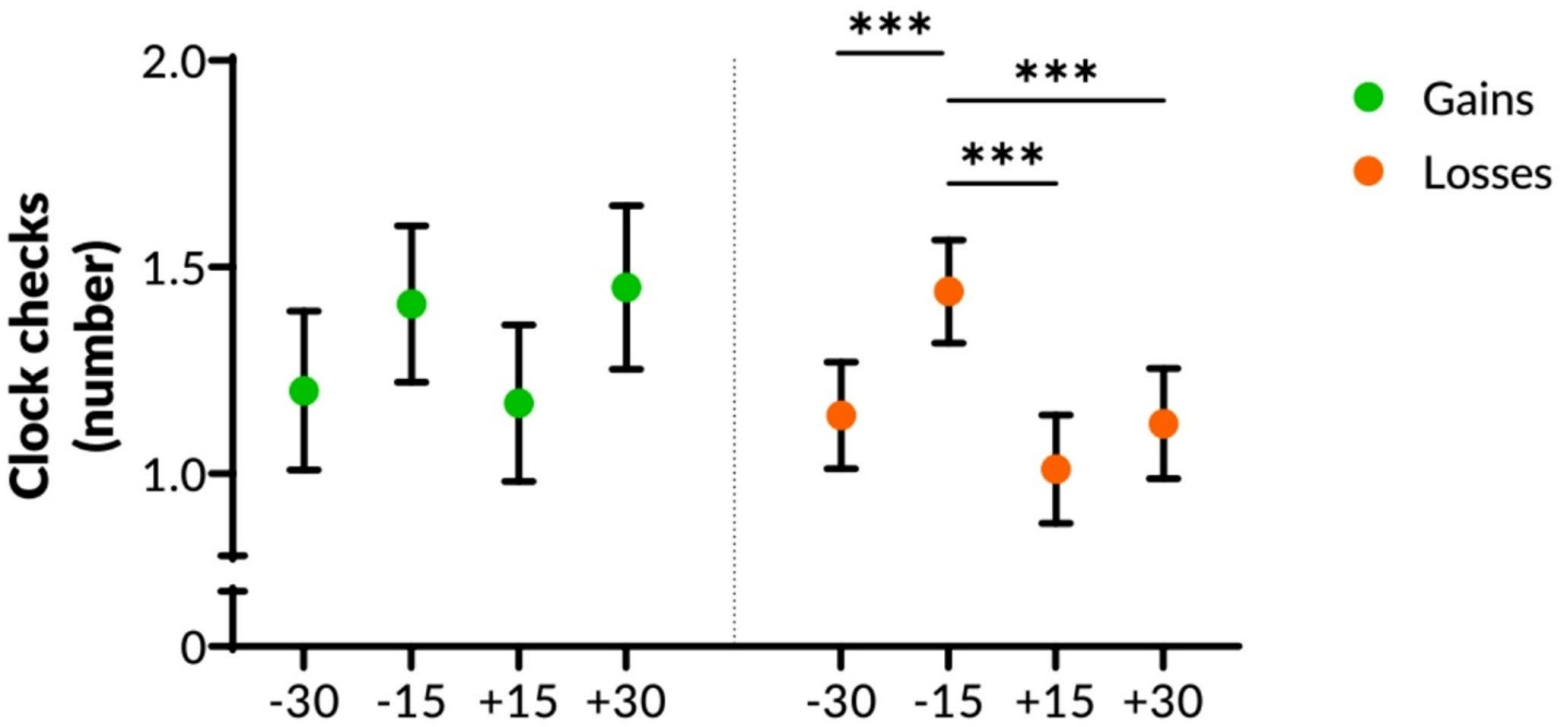
The influence of financial gains or losses on the number of clock checks in a time-based prospective memory task. Participants were able to achieve financial gains or to avoid financial losses, depending on the number of prospective intentions they remembered or missed. We divided the 30s before and after each target time (i.e., prospective intentions) into four intervals (T-30, T-15, T + 15, T + 30), corresponding to 15 or 30s before or after each target. Error bars indicate the standard error of the mean. Significant at *p* <.001^***^

We found no significant effect of incentive type or practice (*p* >.56) but a significant time interval effect (*F*_(3,499.7)_ = 16.50, *p* <.001; Table S10). Participants checked the clock more often in the 15s before each target time than in any other time interval (see Table 3, Tables S11 and S12 in the Supplement). In addition, we found a trend for an interaction between incentive type and time interval (*F*_(3,499.77)_ = 2.52, *p* =.057). Post-hoc tests revealed that more frequent clock-checks in the 15s before target time were evident in the loss condition (Fig. 4), but not in the gain condition (see Tables S13-S15 in Supplement). More frequent clock checking was positively correlated with task accuracy (*r*_s (56)_ = 0.65, *p* <.001). The correlation was stronger for losses (*r*_s (37)_ = 0.71, *p* <.001) than for gains (*r*_s (19)_ = 0.53, *p* =.010), with a medium effect-size difference between the two correlations [33].These results indicate that for clock checking, the type of incentive matters. Although the prospect of avoiding losses had no direct effect on time-based task accuracy, clock monitoring correlated more strongly with task accuracy in the loss condition. This indicates that the prospect of avoiding financial losses may lead to more strategic clock checking, and thus, to more accurate time-based prospective memory, than the prospect of achieving financial gains.

### Ongoing task responses became faster with practice or when participants avoided financial losses

We found a significant effect of time blocks for both task accuracy (*X*^*2*^_(1)_ = 9.14, *p* =.002; Table 3) and response times (*F*_(1,12399)_ = 14.28, *p* <.001; Table 3) as participants became faster– but less accurate– with practice in the ongoing task (see Tables S16-S19 and Fig. 5a/b in the Supplement). In addition, we found a significant effect of incentive type on response times (*F*_(1,127)_ = 5.2, *p* =.024), as participants responded faster in the loss condition than in the gain condition (see Tables S16-S19 and Fig. 5c in the Supplement). We did not find a significant incentive type effect on task accuracy (all *p* >.402) and neither did we find a significant task type effect on task accuracy or response times (all *p* >.122). This indicates that ongoing task accuracy was similar for event-based and time-based prospective memory and was not influenced by the prospect of an incentive. In contrast, participants responded faster when they avoided financial losses. Practicing the 1-back task had a differential effect on performance– it decreased task accuracy but accelerated responses.

### Avoiding financial losses reduced interference in the event-based task, while the prospect of financial gains did so in the time-based task

Adding a prospective memory task to the 1-back task did not lead to interference as participants responded more accurately and faster compared to when they performed only the 1-back task (*X*^*2*^_(1)_ = 49.66, *p* <.001; *F*_(3,15140)_ = 50.29, *p* <.001; see Tables S20-S31 and Fig. 6 in the Supplement). Accuracy increased more strongly (*X*^*2*^_(1)_ = 4.49, *p* =.034) in the loss condition (β = 0.36, SE = 0.05, *p* <.001) than in the gain condition (β = 0.17, SE = 0.06, *p* =.041; see Tables S20-S31 and Fig. 6 in the Supplement). This indicates that avoiding financial losses particularly reduced interference. We found, however, a significant three-way interaction for response times in the 1-back task (*F*_(1,15140)_ = 9.63, *p* =.002). Post-hoc tests revealed that participants responded faster when the 1-back task was embedded in an event-based task and when avoiding financial losses (β = 0.10, SE = 0.02, *p* <.001). When embedded in a time-based task, however, they became faster with the prospect of financial gains (β = 0.07, SE = 0.022, *p* =.014; see Tables S20-S31 and Fig. 7 in Supplement).

## Discussion

Just like other human abilities, prospective memory performance likely improves with practice or when there is an incentive. All participants in our study were able to practice and become familiar with the task. In contrast, incentives differed as one group avoided financial losses, while the other could earn financial gains. Our results confirmed that practice improved event-based prospective memory [22, 23]. Participants gradually answered more accurately and faster as they became familiar with the task. Practicing the task may lead to less demanding (and more automated) retrieval [6]. Automated responses can occur in event-based tasks because intentions are triggered by external events or cues and, hence, these tasks are easier to solve. In addition, it is helpful if prospective memory targets (in our case: pictures of animals) are embedded in an ongoing task that facilitates processing of each target (i.e., a focal task). This was the case in our study as participants had to compare pictures in the ongoing task and, therefore, it was probably easier for them to detect an animal. This is supported by the relatively high accuracy in the ongoing task (~ 65%). In contrast to event-based tasks, retrieval in time-based tasks is difficult to automate which might explain why we did not find an improvement with practice in this task. In time-based tasks, no external event triggers the intention to act, and the clock that could serve as a reminder is only available upon request. Hence, participants need to adopt a strategic, self-initiated retrieval and this is usually difficult for older adults [2]. Another reason for lack of improvement could be a difficult ongoing task. In younger adults, for example, time-based task performance improved particularly with practice when the ongoing task was easy [19, 20, 21]. In our study, the ongoing task was not particularly difficult as participants answered accurately in around 65% of trials. In addition, performance in the ongoing task did not differ between event-based and time-based prospective memory, and we *did* find practice effects in the event-based task. Hence, it was rather the time-based task that was difficult for our participants than the ongoing task. Lack of practice effects may also indicate that participants need more time than ten minutes to adapt to a time-based task (i.e., to find a good strategy to initiate retrieval on their own). To test that hypothesis, future studies could extent time-based tasks to longer durations if they want to test for practice effects.

We also examined whether gain- or loss-related incentives improved performance. In event based prospective memory experiments shorter response times and higher accuracy can both reflect improved performance. Similar to a previous study [13] we observed that loss-related incentives improved event-based task performance while the prospect of financial gains did not. That previous study found more accurate responses with loss-related incentives but did not examine speed of responses, or practice effects. We found speedier responses with loss-related incentives. Time-based prospective memory performance depends on developing a strategy for checking the clock more frequently nearer the time when an action is required. This internally generated strategy improved with loss-related incentives but not with gain-related incentives. In addition, investing in a strategy to check the clock more frequently was associated with improved task accuracy with an incentive. In younger adults, this seems to be different as clock checking was not modulated by loss-related incentives in a previous study [18]. Younger adults are, however, usually better in time estimation [19] and hence, an already optimised clock-checking strategy perhaps cannot improve any further with consequently unchanged response accuracy. Our results further extent previous research as we found decreased ongoing task accuracy in addition to faster responses over time, in particular, when participants avoided financial losses. It has been suggested that older adults focus on accuracy rather than speed [34], but this was not the case in our study. It could be that with practice, participants sacrificed accuracy for the sake of faster responses. Alternatively, they prioritised the prospective memory task over the ongoing task– particularly if they wanted to avoid financial losses.

Our results confirm that loss-related incentives work better to improve performance in older adults than the prospect of financial gains. The exact mechanisms by which incentives enhance performance are not fully understood. According to the motivational-cognitive model [10], there are two different pathways to enhance performance with an incentive in prospective memory tasks. First, incentives can influence how well participants monitor for cues. A focus on cue monitoring may come at the cost of performance in the ongoing task in which participants may then respond slower or less accurate. Our results do not support this. Second, incentives may improve encoding and subsequent maintenance of intentions (e.g., deeper encoding or facilitated access to intentions). This would have no effect on ongoing task performance– as in our study. Hence, with loss-related incentives, our participants may have better, e.g., deeper, encoded prospective intentions. The detection of targets or access to those intentions may thus have been easier during retrieval. 

It is also not fully understood why older adults are more likely to respond to avoiding financial losses than to the prospect of financial gains. Given that old age is a phase of life that is associated with loss and uncertainty [35, 36], decision making in older adults may be more conservative by focussing on maintaining what they already have rather than striving to gain more. In addition, most of our participants were already retired at the time of the experiment. The transition from work to retirement changes the source (and height) of income and this may be associated with financial insecurity. Financial insecurity, combined with other factors typically associated with old age (e.g., illness, disability, frailty) may reduce quality of life [37] and may be perceived as losses. Carver [38] argues that there are two ways in which avoidance motivation promotes action. A person may respond to potential dangers by avoiding threatening stimuli or threatening information, referred to as reactive control. Here, the goal is to reduce anxiety and stress by avoiding any threatening information. With effortful control, a tendency for avoidance may also be overcome but the confrontation of potentially negative information may ultimately lead to a better outcome. Our results indicate that participants used effortful control as they did not avoid their response, but rather responded even faster. Hence, initial avoidance impulses may have led to accelerated actions to reduce or eliminate the threat of losing money. Another explanation may be that financial loss incentives improved alertness or attention, which led to speedier responses, and which promote plasticity that underlies learning.

Our results extend previous research as we show that a shift in motivation due to loss-related incentives influenced both how well older adults remember intentions and how they perform in cognitive tasks in general. When avoiding financial losses, participants focused on doing their best in *both* tasks (1-back and prospective memory), which was not the case when they tried to achieve financial gains. Block and Zakay’s [39] attentional gate model proposed that prospective memory responses become more accurate when more attention is allocated to an estimation of elapsed time. In contrast, task accuracy may decrease when attention is allocated to another tasks (e.g., the ongoing task, in our case a 1-back task). Several studies confirmed this model as they found worse task performance (slower response times or reduced task accuracy) in an ongoing task when prospective intentions were added [5, 40, 41, 42, 43]. In our study, however, participants performed *better* in the ongoing task when prospective intentions were added, and this was particularly the case when they tried to avoid financial losses. Hence, our results indicate that avoiding losses may motivate participants to allocate more attention to both tasks (prospective memory and ongoing task) rather than to focus on one task (i.e., prospective memory or ongoing task). The results were different when participants tried to achieve gains because here, their clock checking was less strategic, possibly because they focused more on improving response times in the ongoing task.

### Limitations

Our study may have several limitations. First, this was an online study. Thus, we do not know how well participants understood the tasks and/or followed instructions. However, task accuracy was similar to other prospective memory studies. We also cannot be certain that participants did not use their own clock rather than the clock available in the task. Second, this study lacks a control group, in which participants perform the task without an incentive involved. However, previous studies clearly showed the effect of an incentive on prospective memory in older adults in comparison to a control group [23]. Our main goal was to compare two different types of incentives for their effect on behaviour. While we cannot be certain that either of these incentives were superior to no incentive, the difference in their behaviour modulating effects suggests that behaviour was indeed different with an incentive than with none. Third, the sample size between conditions was unbalanced. The time-based gains condition had fewer participants than the event-based gains condition. This could have influenced statistical power of our analyses.

## Conclusion

Taken together we show that older healthy adults’ prospective memory performance improved with practice. In addition, we show that incentives, and particularly loss-related incentives, can improve prospective memory or the effects of practice. Hence, incentives or incentivised training may be treatment options for older people should prospective memory decline or to maintain healthy performance levels. However, the type of training, and whether it should be combined with incentives, needs to be adapted to the type of performance outcome. If the goal were to improve speed in an event-based task or to use an efficient strategy in a time-based task, avoiding financial losses may be particularly motivating for older adults. In contrast, striving for accurate responses works better with repeated practice– in combination with incentives to avoid financial losses.

**Table 1:** Sociodemographic characteristics of participants. Mean and standard deviations are shown if not stated otherwise

		Event-based gains	Event-based losses	Time-based gains	Time-based losses
N		43	33	19	37
Age in years		68.5 (5.19)	68.34 (4.20)	65.45 (4.19)	69.4 (5.9)
Sex (f/m)		15/28	11/22	8/11	12/25
Education in years		15.83 (2.21)	15.57 (2.06)	17.05 (1.98)	16.45 (2.36)
Retirement (number)	*Retired*	30	28	12	25
	*Not retired*	10	1	6	8
	*Missing*	3	4	1	4
Monthly income in CHF		5426 (3501)	5235 (2803)	5593 (3994)	6224 (3699)

*Abbreviations*: f/m = number female / number male, CHF = Swiss francs

**Table 2:** Perceived health and donation frequency of participants performing event-based and time-based prospective memory tasks. Participants were able to achieve financial gains or to avoid financial losses

			Event-based gains	Event-based losses	Time-based gains	Time-based losses
Perceived health (in %)		*Very good*	16%	9%	26%	11%
		*Good*	53%	58%	47%	68%
		*Average*	26%	30%	26%	22%
		*Bad*	5%	3%	0%	0%
		*Very bad*	0%	0%	0%	0%
Donation frequency (in %)		*Regularly*	35%	39%	42%	32%
		*Rarely*	42%	39%	47%	43%
		*Never*	16%	9%	5%	14%
		*Missing*	7%	12%	5%	11%

**Table 3:** Means and standard deviations in an event-based or time-based prospective memory task as well as an ongoing task (with or without prospective memory)

	Event-based gains		Event-based losses		Time-based gains		Time-based losses	
	Block 1	Block 2	Block 1	Block 2	Block 1	Block 2	Block 1	Block 2
Task accuracy in %	68.37 (46.61)	73.49 (44.24)	66.06 (47.49)	71.52 (45.27)	29.47 (45.83)	23.15 (42.41)	30.27 (46.07)	32.97 (47.13)
Response time in s	1.81 (0.63)	1.65 (0.48)	1.65 (0.61)	1.42 (0.44)	-	-	-	-
Number of clock checks	-	-	-	-	1.65 (1.12)	1.78 (1.20)	1.48 (0.79)	1.47 (0.72)
Performance in the ongoing task when a prospective memory task was added:								
Task accuracy in %	67.93 (46.69)	66.22 (47.31)	68.87 (46.31)	67.05 (47.01)	69.86 (45.90)	67.29 (46.93)	69.84 (45.89)	67.52 (46.83)
Response time in s	0.96 (0.39)	0.94 (0.36)	0.96 (0.38)	0.93 (0.36)	0.99 (0.40)	0.94 (0.37)	0.95 (0.37)	0.94 (0.37)
Performance in the ongoing task (ongoing task only):								
Task accuracy in %	62.40 (48.46)		60.85 (48.84)		65.79 (47.49)		59.46 (49.12)	
Response time in s	1.00 (0.43)		1.06 (0.45)		1.07 (0.50)		1.00 (0.44)	

Abbreviation: s = seconds

## Electronic supplementary material

Below is the link to the electronic supplementary material.

Supplementary Material 1

## Data Availability

The datasets generated and analysed for this publication are available https://boris-portal.unibe.ch/entities/productd8358c38-5683-4936-a7d0-6bdcb0b8f10c.

## References

[1] Einstein GO, McDaniel MA (1990) Normal aging and prospective memory. J Exp Psychol Learn Mem Cogn 16:717–726. 10.1037//0278-7393.16.4.717 2142956

[2] Craik F (1986) A functional account of age differences in memory. Elsevier North-Holland, Amsterdam

[3] Park DC, Hertzog C, Kidder DP et al (1997) Effect of age on event-based and time-based prospective memory. Psychol Aging 12:314–327. 10.1037/0882-7974.12.2.314 9189992 10.1037//0882-7974.12.2.314

[4] Bastin C, Meulemans T (2002) Are time-based and event-based prospective memory affected by normal aging in the same way? Curr Psychol Lett Behav Brain Cogn 7:105–121

[5] Jäger T, Kliegel M (2008) Time-Based and Event-Based prospective memory across adulthood: underlying mechanisms and differential costs on the ongoing task. J Gen Psychol 135:4–22. 10.3200/GENP.135.1.4-22 18318405 10.3200/GENP.135.1.4-22

[6] McDaniel MA, Einstein GO (2000) Strategic and automatic processes in prospective memory retrieval: a multiprocess framework. Appl Cogn Psychol 14:S127–S144. 10.1002/acp.775

[7] Mcdaniel M, Guynn M, Einstein G, Breneiser J (2004) Cue-Focused and Reflexive-Associative processes in prospective memory retrieval. J Exp Psychol Learn Mem Cogn 30:605–614. 10.1037/0278-7393.30.3.605 15099129 10.1037/0278-7393.30.3.605

[8] Cona G, Bisiacchi PS, Sartori G, Scarpazza C (2016) Effects of cue focality on the neural mechanisms of prospective memory: A meta-analysis of neuroimaging studies. Sci Rep 6:25983. 10.1038/srep25983 27185531 10.1038/srep25983 PMC4868976

[9] Tagliabue CF, Assecondi S, Cristoforetti G, Mazza V (2020) Learning by task repetition enhances object individuation and memorization in the elderly. Sci Rep 10:19957. 10.1038/s41598-020-75297-x 33203888 10.1038/s41598-020-75297-x PMC7673120

[10] Penningroth SL, Scott WDA Motivational-Cognitive Model of Prospective Memory

[11] Peter J, Kliegel M (2018) The age-prospective memory paradox: is it about motivation? Clin Transl Neurosci 2:2514183X18807103. 10.1177/2514183X18807103

[12] Schnitzspahn KM, Ihle A, Henry JD et al (2011) The age-prospective memory-paradox: an exploration of possible mechanisms. Int Psychogeriatr 23:583–592. 10.1017/S1041610210001651 20843394 10.1017/S1041610210001651

[13] Horn SS, Freund AM (2021) How do gain and loss incentives affect memory for intentions across adulthood?? J Gerontol B Psychol Sci Soc Sci 76:711–721. 10.1093/geronb/gbaa140 32877530 10.1093/geronb/gbaa140

[14] Ebner N, Freund A, Baltes P (2006) Developmental changes in personal goal orientation from young to late adulthood: from striving for gains to maintenance and prevention of losses. Psychol Aging 21:664–678. 10.1037/0882-7974.21.4.664 17201488 10.1037/0882-7974.21.4.664

[15] Frank MJ, Kong L (2008) Learning to avoid in older age. Psychol Aging 23:392–398. 10.1037/0882-7974.23.2.392 18573012 10.1037/0882-7974.23.2.392

[16] Cook GI, Rummel J, Dummel S (2015) Toward an Understanding of motivational influences on prospective memory using value-added intentions. Front Hum Neurosci 9. 10.3389/fnhum.2015.00278 10.3389/fnhum.2015.00278 PMC4435068 26042017

[17] Blondelle G, Quaglino V, Gounden Y et al (2024) I won’t forget to do it if it’s important: A multinomial processing tree analysis of social importance and monetary reward on Event-Based prospective memory. J Cogn 7:43. 10.5334/joc.367 38765760 10.5334/joc.367 PMC11100544

[18] Laera G, Brummer J, Hering A et al (2024) The cost of monitoring in time-based prospective memory. Sci Rep 14:2279. 10.1038/s41598-024-52501-w 38280894 10.1038/s41598-024-52501-w PMC10821954

[19] Gan J, Guo Y (2019) The cognitive mechanism of the practice effect of time-Based prospective memory: the role of time Estimation. Front Psychol 10:2780. 10.3389/fpsyg.2019.02780 31866922 10.3389/fpsyg.2019.02780 PMC6909009

[20] Guo Y, Liu P, Huang X (2019) The practice effect on Time-Based prospective memory: the influences of ongoing task difficulty and delay. Front Psychol 10:2002. 10.3389/fpsyg.2019.02002 31555183 10.3389/fpsyg.2019.02002 PMC6722217

[21] Gan J, Guo Y, Wang E (2023) The processing mechanism of repetitive practice affecting Time-Based prospective memory. Behav Sci Basel Switz 13. 10.3390/bs13090750 10.3390/bs13090750 PMC10525505 37754028

[22] Blondelle G, Hainselin M, Gounden Y et al (2016) Regularity effect in prospective memory during aging. Socioaffective Neurosci Psychol 6:31238. 10.3402/snp.v6.31238 10.3402/snp.v6.31238 PMC5091617 27774954

[23] Rose NS, Rendell PG, McDaniel MA et al (2010) Age and individual differences in prospective memory during a virtual week: the roles of working memory, vigilance, task regularity, and cue focality. Psychol Aging 25:595–605. 10.1037/a0019771 20853967 10.1037/a0019771 PMC3065819

[24] Schmidt N, Menéndez-Granda M, Wyss P et al (2024) Financial and prosocial rewards differentially enhance cognition in younger and older healthy adults. Motiv Emot. 10.1007/s11031-024-10092-z

[25] Schmidt N, Haas M, Krebs C et al Clock monitoring is associated with age-related decline in time-based prospective memory. Curr Psychol. 10.1007/s12144-022-03005-1

[26] Whelan R (2008) Effective analysis of reaction time data. Psychol Rec 58:475–482. 10.1007/BF03395630

[27] Berger A, Kiefer M (2021) Comparison of different response time outlier exclusion methods: A simulation study. Front Psychol 12. 10.3389/fpsyg.2021.675558 10.3389/fpsyg.2021.675558 PMC8238084 34194371

[28] Jost L, Jansen P (2022) Using linear mixed models to analyze learning processes within sessions improves detection of treatment effects: an exemplary study of chronometric mental rotation. Methods Psychol 6:100092. 10.1016/j.metip.2022.100092

[29] Salthouse TA (1985) Speed of behavior and its implications for cognition. Handb Psychol Aging 2nd Ed 400–426

[30] Kreidler SM, Ringham BM, Muller KE, Glueck DH (2021) A power approximation for the Kenward and Roger Wald test in the linear mixed model. PLoS ONE 16:e0254811. 10.1371/journal.pone.0254811 34288958 10.1371/journal.pone.0254811 PMC8294572

[31] Kuznetsova A, Brockhoff PB, Christensen RHB (2017) LmerTest package: tests in linear mixed effects models. J Stat Softw 82. 10.18637/jss.v082.i13

[32] Bates D, Mächler M, Bolker B, Walker S (2015) Fitting linear Mixed-Effects models using lme4. J Stat Softw 67. 10.18637/jss.v067.i01

[33] Cohen J (2013) Statistical power analysis for the behavioral sciences. routledge

[34] Starns JJ, Ratcliff R (2010) The effects of aging on the speedâ€accuracy compromise: boundary optimality in the diffusion model. Psychol Aging 25:377–390. 10.1037/a0018022 20545422 10.1037/a0018022 PMC2896207

[35] Pitt B (1998) Loss in late life. BMJ 316:1452–1454. 10.1136/bmj.316.7142.1452 9572763 10.1136/bmj.316.7142.1452 PMC1113122

[36] Baltes PB, Lindenberger U, Staudinger UM (1998) Life-span theory in developmental psychology. Handb Child Psychol Theor Models Hum Dev 1(5):1029–1143

[37] Eurostat (2020) Ageing Europe—Looking at the Lives of Older People in the EU—2020 Edition. https://ec.europa.eu/eurostat/web/products-statistical-books/-/ks-02-20-655

[38] Carver CS (2005) Impulse and constraint: perspectives from personality psychology, convergence with theory in other areas, and potential for integration. Personality Social Psychol Rev 37:90–101. 10.1207/s15327957pspr0904_2 10.1207/s15327957pspr0904_2 16223354

[39] Block RA, Zakay D (2006) Prospective Remembering Involves Time Estimation and Memory Processes. World Scientific Publishing Co. 10.1142/9789812707123_0002

[40] Cona G, Arcara G, Tarantino V, Bisiacchi P (2012) Age-related differences in the neural correlates of remembering time-based intentions. Neuropsychologia 50:2692–2704. 10.1016/j.neuropsychologia.2012.07.033 22841994 10.1016/j.neuropsychologia.2012.07.033

[41] Cottini M, Meier B (2020) Prospective memory monitoring and aftereffects of deactivated intentions across the lifespan. Cogn Dev

[42] Marsh RL, Hicks JL, Cook GI (2006) Task interference from prospective memories covaries with contextual associations of fulfilling them. Mem Cognit 34:1037–1045. 10.3758/BF03193250 17128602 10.3758/bf03193250

[43] Hicks JL, Marsh RL, Cook GI (2005) Task interference in time-based, event-based, and dual intention prospective memory conditions. J Mem Lang 53:430–444. 10.1016/j.jml.2005.04.001

