# Supplementary material for: Incentives and practice improve prospective memory performance in older adults
Source: Aging Clin Exp Res. 2025 Jul 9;37(1):212. doi: 10.1007/s40520-025-03127-z (PMC12241248; doi:10.1007/s40520-025-03127-z)

**Incentives and practice improve prospective memory in older adults**

Marta Menéndez-Granda^1,2^, Nadine Schmidt^1,2^, Michael Orth^1^, Sebastian Horn^3^, Matthias Kliegel^4,5,6^, Jessica Peter^1^

1 University Hospital of Old Age Psychiatry and Psychotherapy, University of Bern, Switzerland

2 Graduate School for Health Sciences, University of Bern, Switzerland

3 Department of Psychology, University of Zurich, Switzerland

4 Faculty of Psychology and Educational Sciences, University of Geneva, Switzerland

5 Centre for the Interdisciplinary Study of Gerontology and Vulnerability, University of Geneva, Switzerland

6 Swiss Centre of Expertise in Life Course Research, LIVES Centre, Lausanne and Geneva, Switzerland

Corresponding Author

Jessica Peter

University Hospital of Old Age Psychiatry and Psychotherapy, Bern University, Switzerland Bolligenstrasse 111

CH-3000 Bern 60

Phone: +41 58 630 89 03

Mail: [jessica.peter@unibe.ch](mailto:jessica.peter@unibe.ch)

**Supplementary material**

## **Statistical analysis**

We first examined differences in sociodemographic variables between four conditions: (1) Time-based prospective memory – losses, (2) Event-based prospective memory – losses, (3) Time-based prospective memory – gains (4) Event-based prospective memory – gains. We used Kruskal-Wallis’s test to investigate whether there were differences regarding age, education, or monthly income between different conditions, as these variables were not normally distributed. To test for differences in sex distribution, retirement status and perceived health, we used Chi-Square test. Finally, we calculated the predictive values for each factor and their interactions in fitted models, and we also used post-hoc tests for significant factors and interactions that were reported in the main manuscript.

### **Group comparison of sociodemographic variables**

All conditions were comparable regarding age, sex, education, income, retirement, perceived health, and donation frequency (Table S1).

**Table S1** Comparison of sociodemographic variables between all four conditions.

| Age in years | χ2 (3) = 6.85, *p* = 0.077 |
| --- | --- |
| Sex (f/m) | χ2 (3) = 0.57, *p* = 0.903 |
| Education in years | χ2 (3) = 7.17, *p* = 0.067 |
| Monthly income in CHF | χ2 (3) = 0.99, *p* = 0.803 |
| Retirement status | χ2 (3) = 0.41, *p* = 0.053 |
| Perceived health | χ2 (9) = 7.01, *p* = 0.636 |
| Donation frequency | χ2 (6) = 2.15, *p* = 0.905 |

*Abbreviation*: f/m = number female / number male

### **Results of predicted accuracy and response times, as well as post-hoc tests for significant factors or interactions**

#### Prospective memory task accuracy

We found no main effect of incentive type, or blocks of time. We found, however, a significant main effect of task type, as well as significant interactions between prospective memory task type*blocks of time and incentive type*blocks of time (Table S2). For significant factors or interactions, post-hoc comparisons and contrasts were conducted (see below).

**Table S2** Results of mixed effects models predicting prospective memory task accuracy.

|  | Prospective memory trial accuracy | | |
| --- | --- | --- | --- |
|  | *df* | *χ^2^*-value | *p*-value |
| Prospective memory task type | 1 | 19.960 | **< 0.001** |
| Incentive type | 1 | 0.006 | 0.941 |
| Blocks of time | 1 | 3.466 | 0.056 |
| Prospective memory task type*Incentive type | 1 | 0.140 | 0.708 |
| Prospective memory task type*Blocks of time | 1 | 7.157 | **0.011** |
| Incentive type*Blocks of time | 1 | 3.166 | 0.075 |
| Prospective memory task type*Incentive type*Blocks of time | 1 | 0.421 | 0.516 |

*Abbreviation*: *df* = degrees of freedom.

###### *Factor: Task type*

The probability of responding correctly was significantly higher in event-based prospective memory trials than in time-based prospective memory trials (*X^2^* _(1, 132)_ = 19.960, *p* < .001; Table S3).

**Table S3** Estimated mean probabilities of responding accurately, depending on task type.

|  |  |  | **95%-Confidence Level** | |
| --- | --- | --- | --- | --- |
| **Task type** | **Probability** | **SE** | **lower** | **upper** |
| Event-based task | 0.945 | 0.040 | 0.790 | 0.987 |
| Time-based task | 0.037 | 0.033 | 0.006 | 0.190 |

*Abbreviation*: SE = standard error.

###### *Interaction: Task type * Blocks of time*

In, the event-based task, participants were more accurate in block 2 than in block 1 (Table S4-S6).

**Table S4** Estimated mean probabilities, depending on task type and blocks of time.

|  |  |  |  | **95%-Confidence Level** | |
| --- | --- | --- | --- | --- | --- |
| **Task type** | **Blocks of time** | **Probability** | **SE** | **lower** | **upper** |
| Event-based task | 1 | 0.911 | 0.063 | 0.690 | 0.980 |
|  | 2 | 0.967 | 0.026 | 0.856 | 0.993 |
| Time-based task | 1 | 0.041 | 0.036 | 0.007 | 0.208 |
|  | 2 | 0.034 | 0.031 | 0.006 | 0.180 |

*Abbreviation*: SE = standard error.

**Table S5** Contrasts for probabilities in block 1 compared to 2, depending on task type.

| **Task type** | **Contrast** | **Estimate** | **SE** | **z-value** | ***p*-value** |
| --- | --- | --- | --- | --- | --- |
| Event-based task | 1 – 2 | -1.04 | 0.349 | -2.983 | **0.015** |
| Time-based task |  | 0.191 | 0.317 | 0.604 | 0.931 |

*Abbreviation*: SE = standard error.

**Table S6** Contrasts for probabilities in the event-based task compared to the time-based task, depending on blocks of time.

| **Blocks of time** | **Contrast** | **Estimate** | **SE** | **z-value** | ***p*-value** |
| --- | --- | --- | --- | --- | --- |
| 1 | Event – Time-based | 5.491 | 1.313 | 4.182 | **<0.001** |
| 2 |  | 6.723 | 1.350 | 4.981 | **<0.001** |

*Abbreviation*: SE = standard error.


#### Event-based prospective memory – response times

We found a significant main effect of incentive type and blocks of time. The interaction was not significant. For significant factors, post-hoc comparisons and contrasts were conducted.

**Table S7** Results of mixed effects models predicting event-based response times.

|  |  | Log (response times) | | | |
| --- | --- | --- | --- | --- | --- |
|  | *df1* | | *df2* | *F-*value | *p*-value |
| Incentive type | 1 | | 55.95 | 5.575 | **0.022** |
| Blocks of time | 1 | | 442.95 | 24.181 | **<0.001** |
| Incentive type*Blocks of time | 1 | | 442.73 | 0.124 | 0.725 |

*Abbreviations*: *df1* = nominator degrees of freedom, *df2* = denominator degrees of freedom.

###### *Factor: Incentive type*

Participants answered faster in the loss condition compared to the gain condition.

**Table S8** Back-transformed mean response times for participants in the gains or losses condition.

|  |  |  | **95%-Confidence Level** | |
| --- | --- | --- | --- | --- |
| **Incentive type** | **Estimated mean** | **SE** | **lower** | **upper** |
| Gains | 1.70 | 0.078 | 1.55 | 1.86 |
| Losses | 1.43 | 0.080 | 1.28 | 1.60 |

*Abbreviation*: SE = standard error.

###### *Factor: Blocks of time*

Participants became faster with practice.

**Table S9** Back-transformed mean response times, depending on blocks of time.

|  |  |  | **95%-Confidence Level** | |
| --- | --- | --- | --- | --- |
| **Blocks of time** | **Estimated mean** | **SE** | **lower** | **upper** |
| 1 | 1.63 | 0.061 | 1.51 | 1.75 |
| 2 | 1.49 | 0.055 | 1.38 | 1.61 |

*Abbreviation*: SE = standard error.

#### Time-based prospective memory – clock checking

We found no significant main effect of incentive type or blocks of time. We found, however, a significant time interval effect and a trend towards a significant interaction between incentive type and time interval. For significant factors or interactions, post-hoc comparisons and contrasts were conducted (see below).

**Table S10** Results of mixed effects models predicting clock monitoring.

|  |  | | Clock monitoring | | | |
| --- | --- | --- | --- | --- | --- | --- |
|  | | df1 | | df2 | *F-*value | *p*-value |
| Incentive type | | 1 | | 36.42 | 0.221 | 0.641 |
| Time interval | | 3 | | 499.69 | 16.474 | **<0.001** |
| Blocks of time | | 1 | | 497.41 | 0.095 | 0.759 |
| Incentive type*Time interval | | 3 | | 499.77 | 2.523 | 0.057 |
| Incentive type*Blocks of time | | 1 | | 495.98 | 2.922 | 0.088 |
| Time interval*Blocks of time | | 3 | | 496.34 | 0.945 | 0.419 |
| Incentive type*Time interval*Blocks of time | | 3 | | 495.35 | 1.441 | 0.230 |

*Abbreviation*: df = degrees of freedom.

###### *Factor: Time interval*

Participants checked the clock more frequently 15 seconds before each target time.

**Table S11** Estimated mean number of clock checks, depending on time interval.

|  |  |  |  | **95%-Confidence Level** | |
| --- | --- | --- | --- | --- | --- |
| **Time interval** | **Estimated mean** | **SE** | **df** | **lower** | **upper** |
| T - 30 | 1.17 | 0.116 | 43 | 0.934 | 1.40 |
| T - 15 | 1.42 | 0.113 | 39.5 | 1.195 | 1.65 |
| T + 15 | 1.09 | 0.115 | 42.1 | 0.858 | 1.32 |
| T + 30 | 1.29 | 0.119 | 48.5 | 0.934 | 1.40 |

*Abbreviations*: SE = standard error, df = degrees of freedom.

**Table S12** Contrasts for number of clock checks, depending on time interval.

| **Contrast** | **Estimate** | **SE** | **t-value** | ***p*-value** |
| --- | --- | --- | --- | --- |
| T - 15 – T - 30 | 0.256 | 0.061 | 4.216 | **<0.001** |
| T - 15 – T + 15 | 0.334 | 0.064 | 5.235 | **<0.001** |
| T - 15 – T + 30 | 0.138 | 0.069 | 2.006 | 0.187 |
| T - 30 – T + 15 | 0.078 | 0.069 | 1.131 | 0.671 |
| T - 30 – T + 30 | -0.119 | 0.070 | -1.701 | 0.324 |
| T + 15 – T + 30 | -0.197 | 0.074 | -2.651 | **0.041** |

*Abbreviation*: SE = standard error.

###### *Interaction: Incentive type * Time interval*

Participants in the loss condition checked the clock more frequently when target times approached, while this was not the case for participants in the gain condition.

**Table S13** Estimated mean number of clock checks, depending on time interval and incentive type.

|  |  |  |  |  | **95%-Confidence Level** | |
| --- | --- | --- | --- | --- | --- | --- |
| **Incentive type** | **Time interval** | **Estimated mean** | **SE** | **df** | **lower** | **upper** |
| Gains | T - 30 | 1.20 | 0.192 | 42.9 | 0.809 | 1.58 |
|  | T - 15 | 1.41 | 0.189 | 39.9 | 1.028 | 1.79 |
|  | T + 15 | 1.17 | 0.189 | 40.2 | 0.790 | 1.55 |
|  | T + 30 | 1.45 | 0.198 | 48.4 | 1.052 | 1.85 |
| Losses | T - 30 | 1.14 | 0.129 | 43.2 | 0.878 | 1.40 |
|  | T - 15 | 1.44 | 0.125 | 38.5 | 1.185 | 1.69 |
|  | T + 15 | 1.01 | 0.131 | 46.6 | 0.744 | 1.27 |
|  | T + 30 | 1.12 | 0.133 | 48.7 | 0.856 | 1.39 |

*Abbreviations*: SE = standard error, df = degrees of freedom.

**Table S14** Contrasts for number of clock checks in the gain condition compared to the losses condition, depending on time interval.

| **Time interval** | **Contrast** | **Estimate** | **SE** | **df** | **t-value** | ***p*-value** |
| --- | --- | --- | --- | --- | --- | --- |
| T - 30 | Gains – Losses | 0.058 | 0.231 | 43 | 0.249 | 1.000 |
| T - 15 |  | -0.029 | 0.226 | 39.5 | -0.129 | 1.000 |
| T + 15 |  | 0.163 | 0.230 | 42.1 | 0.707 | 0.996 |
| T + 30 |  | 0.328 | 0.239 | 48.5 | 1.375 | 0.864 |

*Abbreviations*: SE = standard error, df = degrees of freedom.

**Table S15** Contrasts for number of clock checks in the different time intervals, depending on incentive type.

| **Incentive** | **Contrast** | **Estimate** | **SE** | **df** | **t-value** | ***p*-value** |
| --- | --- | --- | --- | --- | --- | --- |
|  | T - 15 – T - 30  T - 15 – T + 15 | 0.213 | 0.103 | 494.1 | 2.060 | 0.443 |
| Gains |  | 0.238 | 0.107 | 499.5 | 2.236 | 0.331 |
|  | T - 15 – T + 30 | -0.041 | 0.116 | 496.5 | -0.353 | 1.000 |
|  | T - 30 – T + 15 | 0.025 | 0.115 | 501.7 | 0.219 | 1.000 |
|  | T - 30 – T + 30 | -0.254 | 0.117 | 491.8 | -2.169 | 0.373 |
|  | T + 15 –T + 30 | -0.279 | 0.124 | 502.7 | -2.246 | 0.326 |
|  | T - 15 – T - 30 | 0.300 | 0.064 | 494.1 | 4.676 | **<0.001** |
|  | T - 15 – T + 15 | 0.430 | 0.070 | 497.6 | 6.112 | **<0.001** |
| Losses | T - 15 – T + 30 | 0.316 | 0.074 | 499.5 | 4.298 | **<0.001** |
|  | T - 30 – T + 15 | 0.130 | 0.075 | 495.3 | 1.732 | 0.666 |
|  | T - 30 – T + 30 | 0.016 | 0.076 | 494.6 | 0.213 | 1.000 |
|  | T + 15 – T + 30 | -0.114 | 0.081 | 496.9 | -1.409 | 0.853 |

*Abbreviations*: SE = standard error, df = degrees of freedom.

#### Ongoing task performance during the prospective memory task – accuracy and response times

In terms of accuracy, we found a significant main effect of blocks of time. No other factor or any interaction reached significance. For response times, two main effects became significant: incentive type and blocks of time. For significant factors or interactions, post-hoc comparisons and contrasts were conducted.

**Table S16** Results of mixed effects models predicting trial accuracy or response times in the ongoing task when the prospective memory task was added.

|  | Trial accuracy | | |  | Response time | | | |
| --- | --- | --- | --- | --- | --- | --- | --- | --- |
|  | *df* | *χ^2^*-value | *p*-value |  | *df*_1_ | *df*_2_ | *F*-value | *p*-value |
| Task type | 1 | 2.388 | 0.122 |  | 1 | 126.4 | 1.633 | 0.203 |
| Incentive type | 1 | 0.702 | 0.402 |  | 1 | 127.0 | 5.200 | **0.024** |
| Blocks of time | 1 | 9.142 | **0.002** |  | 1 | 12399.0 | 14.280 | **0.000** |
| Task type*Incentive type | 1 | 0.305 | 0.581 |  | 1 | 126.5 | 0.192 | 0.661 |
| Task type*Blocks of time | 1 | 0.251 | 0.617 |  | 1 | 12399.5 | 0.371 | 0.542 |
| Incentive type*Blocks of time | 1 | 0.000 | 0.992 |  |  | 12401.5 | 0.065 | 0.799 |
| Task type*Incentive type*Blocks of time | 1 | 0.018 | 0.893 |  | 1 | 12398.2 | 1.266 | 0.261 |

*Abbreviations*: df = degrees of freedom, *df*1 = nominator degrees of freedom; *df*2 = denominator degrees of freedom.

###### *Accuracy –* *Factor: Blocks of time*

Task accuracy in the ongoing task decreased over time.

**Table S17** Estimated mean probabilities, depending on blocks of time.

|  |  |  | **95%-Confidence Level** | |
| --- | --- | --- | --- | --- |
| **Blocks of time** | **Probability** | **SE** | **lower** | **upper** |
| 1 | 0.691 | 0.001 | 0.682 | 0.701 |
| 2 | 0.670 | 0.005 | 0.660 | 0.680 |

*Abbreviation*: SE = standard error.

###### *Response times – Factor: Incentive type*

Participants answered faster in the loss condition compared to the gain condition.

**Table S18** Back-transformed mean response times for participants that tried to achieve financial gains or to avoid financial losses.

|  |  |  | **95%-Confidence Level** | |
| --- | --- | --- | --- | --- |
| **Incentive type** | **Estimated mean** | **SE** | **lower** | **upper** |
| Gains | 0.859 | 0.003 | 0.852 | 0.866 |
| Losses | 0.848 | 0.003 | 0.842 | 0.854 |

*Abbreviation*: SE = standard error.

###### *Response times –* *Factor: Blocks of time*

Participants became faster with practice.

**Table S19** Back-transformed means response times, depending on blocks of time.

|  |  |  | **95%-Confidence Level** | |
| --- | --- | --- | --- | --- |
| **Blocks of time** | **Estimated mean** | **SE** | **lower** | **upper** |
| 1 | 0.897 | 0.003 | 0.890 | 0.904 |
| 2 | 0.878 | 0.003 | 0.871 | 0.885 |

*Abbreviation*: SE = standard error.

**Fig.** **5** **Ongoing task performance when a prospective memory task was added**. Task performance over time was divided into 2 blocks of 5 minutes each. a) Task accuracy, b) Response time, c) Response time when avoiding losses or achieving gains. Error bars indicate the standard error of the mean. Significant at p < 0.05*, p < 0.01**, or p < 0.001***.


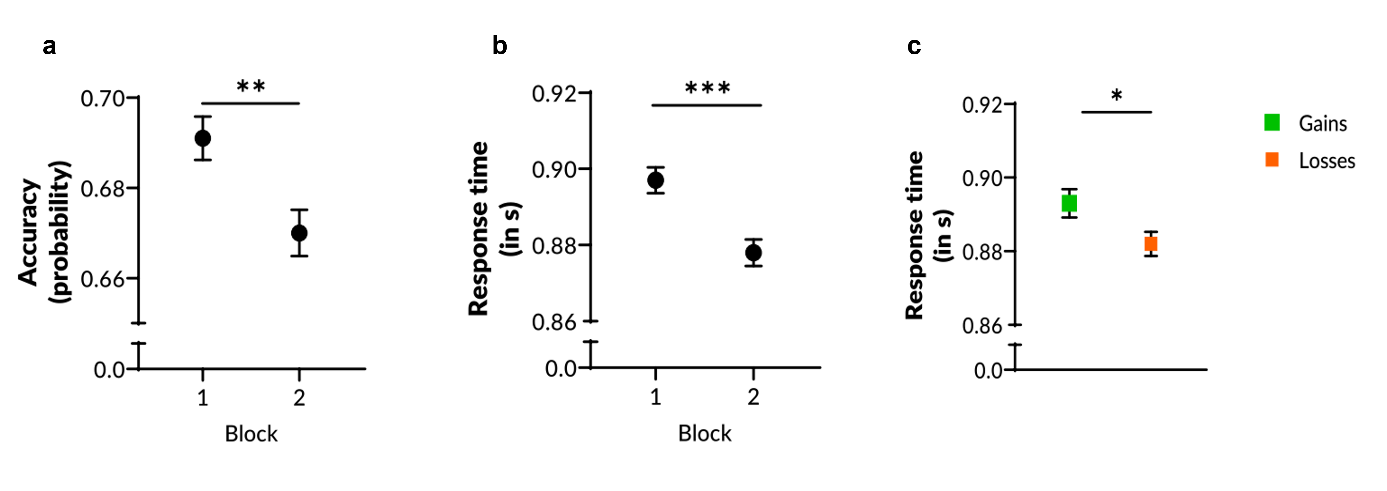


#### Performance in the ongoing only block or during the prospective memory block – accuracy and response times

In terms of accuracy, we found a significant main effect of ongoing task condition (ongoing only or ongoing plus prospective memory). In addition, the interaction with incentive type was significant. Regarding response times, a significant main effect of ongoing task condition was found, as well as two significant interactions: prospective memory task type*ongoing task condition and prospective memory task type*incentive type*ongoing task condition. For significant factors or interactions, post-hoc comparisons and contrasts were conducted.

**Table S20** Results of mixed effects models predicting trial accuracy or response times in an ongoing task.

|  | Trial accuracy | | |  | Response time | | | |
| --- | --- | --- | --- | --- | --- | --- | --- | --- |
|  | *df* | *χ^2^*-value | *p*-value |  | *df*_1_ | *df*_2_ | *F*-value | *p*-value |
| Task type | 1 | 1.692 | 0.193 |  | 1 | 15140 | 0.828 | 0.363 |
| Incentive type | 1 | 0.000 | 0.996 |  | 1 | 15140 | 3.211 | 0.073 |
| Ongoing task condition | 1 | 49.660 | **<0.001** |  | 1 | 15140 | 50.294 | **<0.001** |
| Task type*Incentive type | 1 | 0.713 | 0.398 |  | 1 | 15140 | 4.137 | **0.042** |
| Task type*Ongoing task condition | 1 | 0.065 | 0.799 |  | 1 | 15140 | 2.504 | 0.114 |
| Incentive type* Ongoing task condition | 1 | 4.487 | **0.034** |  |  | 15140 | 1.587 | 0.207 |
| Task type*Incentive type*Ongoing task condition | 1 | 1.042 | 0.307 |  | 1 | 15140 | 9.631 | **0.002** |

*Abbreviations*: df = degrees of freedom, *df*1 = nominator degrees of freedom; *df*2 = denominator degrees of freedom.

###### *Accuracy –* *Factor: Ongoing task condition*

The probability of responding accurately in the ongoing task was higher for the prospective memory block, in which participants had to do an ongoing task and a prospective memory task simultaneously.

**Table S21** Estimated means probabilities, depending on ongoing task condition.

|  |  | |  | | **95%-Confidence Level** | |
| --- | --- | --- | --- | --- | --- | --- |
| **Ongoing task condition** | | **Probability** | | **SE** | **lower** | **upper** |
| Ongoing only | | 0.622 | | 0.009 | 0.603 | 0.64 |
| Ongoing + prospective memory | | 0.682 | | 0.004 | 0.674 | 0.69 |

*Abbreviation*: SE = standard error.

###### *Accuracy –* *Interaction: Incentive type * Ongoing task condition*

The difference in accuracy between ongoing only and ongoing plus prospective memory task was greater for the losses condition than for the gains condition.

**Table S22** Estimated mean probabilities, depending on ongoing task condition and incentive type.

|  |  |  |  | **95%-Confidence Level** | |
| --- | --- | --- | --- | --- | --- |
| **Incentive type** | **Ongoing task condition** | **Probability** | **SE** | **lower** | **upper** |
| Gains | Ongoing only | 0.642 | 0.014 | 0.614 | 0.669 |
|  | Ongoing + PM | 0.679 | 0.006 | 0.667 | 0.691 |
| Losses | Ongoing only | 0.602 | 0.012 | 0.577 | 0.626 |
|  | Ongoing + PM | 0.684 | 0.005 | 0.674 | 0.695 |

*Abbreviations*: SE = standard error, PM = prospective memory task.

**Table S23** Contrasts for probabilities in the ongoing only condition compared to the ongoing plus prospective memory task condition, depending on incentive type.

| **Incentive type** | **Contrast** | **Estimate** | **SE** | **z-value** | ***p*-value** |
| --- | --- | --- | --- | --- | --- |
| Gains | OG only – OG + PM | -0.168 | 0.064 | -2.640 | **0.041** |
| Losses |  | -0.360 | 0.054 | -6.625 | **<0.001** |

*Abbreviations*: SE = standard error, OG = ongoing task, PM = prospective memory.

**Table S24** Contrasts for probabilities in the gains condition compared to the losses condition, depending on the ongoing task condition.

| **Ongoing task condition** | **Contrast** | **Estimate** | **SE** | **z-value** | ***p*-value** |
| --- | --- | --- | --- | --- | --- |
| Ongoing only | Gains – Losses | 0.168 | 0.080 | 2.114 | 0.149 |
| Ongoing + prospective memory |  | -0.023 | 0.038 | -0.592 | 0.935 |

*Abbreviations*: SE = standard error.

**Fig. 6** **Accuracy in an ongoing task alone (OG) or when a prospective memory task was added (OG + PM)**. Participants were able to achieve financial gains or to avoid financial losses, depending on the number of prospective intentions they remembered or missed. Error bars indicate the standard error of the mean. Significant at *p* < .05^*^ or *p* < .001^***^.


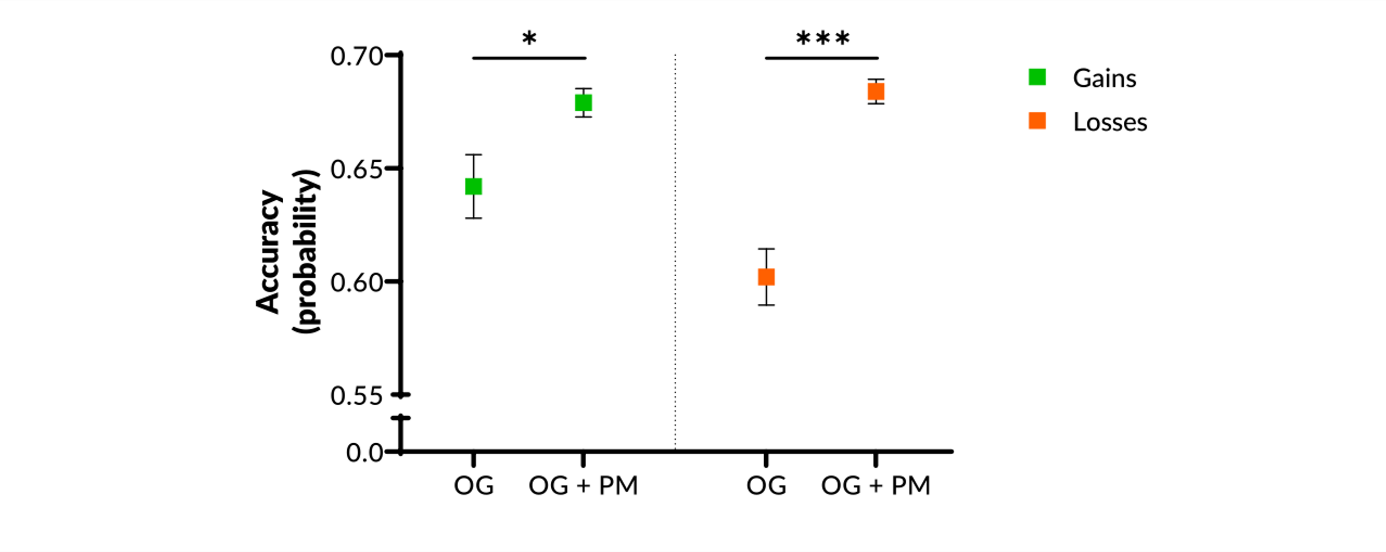


###### *Response times –* *Factor: Ongoing task condition*

Participants responded faster in the ongoing plus prospective memory task than in the ongoing only task.

**Table S25** Back-transformed mean response times in participants that tried to achieve financial gains or to avoid financial losses.

|  |  | |  | | **95%-Confidence Level** | |
| --- | --- | --- | --- | --- | --- | --- |
| **Ongoing task condition** | | **Estimated mean** | | **SE** | **lower** | **upper** |
| Ongoing only | | 0.952 | | 0.008 | 0.937 | 0.968 |
| Ongoing + prospective memory | | 0.894 | | 0.003 | 0.888 | 0.899 |

*Abbreviations*: SE = standard error.

###### *Response times –* *Interaction: Prospective memory task type*Incentive type*

Participants answered faster in the ongoing task during time-based prospective memory in the loss condition, but not in the ongoing task during event-based prospective memory. In addition, participants answered faster in the ongoing task during time-based prospective memory compared to ongoing during event-based prospective memory but only in the loss condition.

**Table S26** Back-transformed mean response times, depending on incentive type and prospective memory task type.

|  |  |  |  | **95%-Confidence Level** | |
| --- | --- | --- | --- | --- | --- |
| **Incentive type** | **PM task type** | **Estimated mean** | **SE** | **lower** | **upper** |
| Gains | Event-based | 0.912 | 0.007 | 0.899 | 0.926 |
|  | Time-based | 0.941 | 0.011 | 0.921 | 0.961 |
| Losses | Event-based | 0.934 | 0.008 | 0.919 | 0.950 |
|  | Time-based | 0.904 | 0.007 | 0.889 | 0.918 |

*Abbreviations*: SE = standard error, PM = prospective memory.

**Table S27** Contrasts for ongoing task response times in the gains condition compared to the losses condition, depending on task type.

| **Task type** | **Contrast** | **Estimate** | **SE** | **t-value** | ***p*-value** |
| --- | --- | --- | --- | --- | --- |
| Event-based | Gains – Losses | -0.024 | 0.011 | -2.131 | 0.143 |
| Time-based |  | 0.040 | 0.014 | 2.993 | **0.014** |

*Abbreviation*: SE = standard error.

**Table S28** Contrasts for ongoing task response times in the event-based task compared to the time-based task, depending on incentive type.

| **Incentive type** | **Contrast** | **Estimate** | **SE** | **t-value** | ***p*-value** |
| --- | --- | --- | --- | --- | --- |
| Gains | Event-based – Time-based | -0.031 | 0.013 | -2.360 | 0.084 |
| Losses |  | 0.033 | 0.011 | 2.856 | **0.022** |

*Abbreviation*: SE = standard error.

###### *Response times –* *Interaction: Prospective memory task type*Incentive type*Ongoing task condition*

Participants responded faster when performing both tasks simultaneously for the event-based task in the loss condition or the time-based task in the gain condition. That was not the case for the other two conditions: time-based task losses or event-based task gains.

**Table S29** Back-transformed mean response times, depending on ongoing task condition, incentive type, and task type.

|  |  |  |  |  | **95%-Confidence Level** | |
| --- | --- | --- | --- | --- | --- | --- |
| **OG** | **Incentive** | **Task type** | **Estimated mean** | **SE** | **lower** | **upper** |
| OG only | Gains | Event-based | 0.988 | 0.014 | 0.962 | 1.015 |
|  |  | Time-based | 1.036 | 0.021 | 0.996 | 1.077 |
|  | Losses | Event-based | 0.998 | 0.011 | 0.977 | 1.019 |
|  |  | Time-based | 0.974 | 0.015 | 0.945 | 1.003 |
| OG + PM | Gains | Event-based | 0.948 | 0.005 | 0.938 | 0.958 |
|  |  | Time-based | 0.962 | 0.008 | 0.947 | 0.977 |
|  | Losses | Event-based | 0.939 | 0.006 | 0.928 | 0.950 |
|  |  | Time-based | 0.944 | 0.005 | 0.933 | 0.954 |

*Abbreviations*: SE = standard error, OG = ongoing task, PM = prospective memory.

**Table S30** Contrasts for response times in the ongoing only task compared to the ongoing plus prospective memory task, depending on task type and incentive type.

| **Task type** | **Incentive** | **Contrast** | **Estimate** | **SE** | **t-value** | ***p*-value** |
| --- | --- | --- | --- | --- | --- | --- |
| Event-based | Gains | OG only – OG+PM | 0.041 | 0.015 | 2.794 | 0.096 |
|  | Losses |  | 0.106 | 0.017 | 6.332 | **<0.001** |
| Time-based | Gains |  | 0.074 | 0.022 | 3.420 | **0.014** |
|  | Losses |  | 0.031 | 0.016 | 1.932 | 0.528 |

*Abbreviations*: SE = standard error, OG = ongoing task, PM = prospective memory task.

**Table S31** Contrasts for response times, depending on task type, incentive type, and ongoing task condition.

| **Task type** | **Contrast 1** | **Contrast 2** | **estimate** | **SE** | **t-value** | ***p*-value** |
| --- | --- | --- | --- | --- | --- | --- |
| Event-based | Gains – Losses | OG only – OG+PM | 0.050 | 0.015 | 3.358 | **0.017** |
| Time-based |  |  | 0.093 | 0.021 | 4.452 | **0.000** |

*Abbreviations*: SE = standard error, OG = ongoing task, PM = prospective memory task.

**Fig. 7** **Response times in an ongoing only task (OG) or when a prospective memory task was added (OG + PM).** Participants were able to achieve financial gains or to avoid financial losses, depending on the number of prospective intentions they remembered or missed. Error bars indicate the standard error of the mean. Significant at *p* < .05^*^ or *p* < .001^***^.


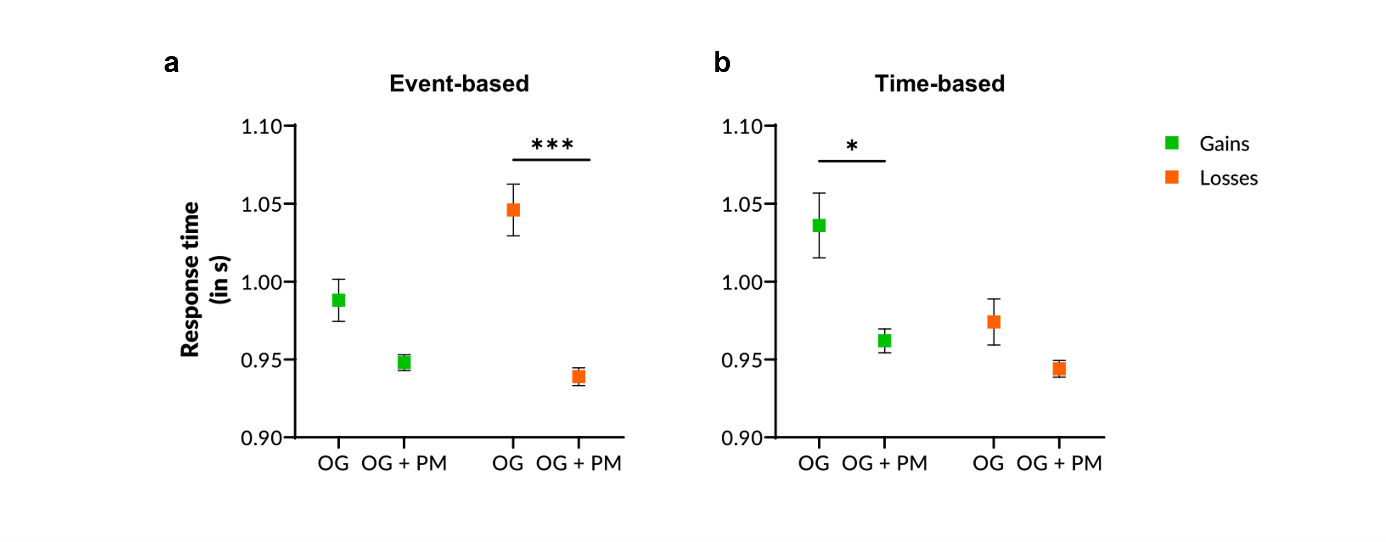

Supplement: Supplementary file 1 — Supplementary Material 1 [file 40520_2025_3127_MOESM1_ESM.docx]
